# Surgical Repair of Achilles Tendon Rupture by Turn-O-Plasty: A Case Report

**DOI:** 10.7759/cureus.29058

**Published:** 2022-09-11

**Authors:** Sarthak Gupta, Ankur Salwan, Gajanan L Pisulkar, Amit Saoji, Shounak Taywade

**Affiliations:** 1 Department of Orthopaedic Surgery, Jawaharlal Nehru Medical College, Datta Meghe Institute of Medical Sciences, Wardha, IND

**Keywords:** weak plantar flexion, positive thompson test, calcaneum, watershed, tendo achilles rupture, suturedisc, turnoplasty

## Abstract

Achilles tendon rupture has been a difficult problem for surgeons, especially in older patients, since tendon strength and flexibility are significantly diminished compared to young people. The Achilles tendon endures the highest tensile stresses in the body while running, leaping, and skipping, with tensile loads up to 10 times body weight. There are many treatment options for Achilles tendon repair, including open surgery, percutaneous repair, and ultrasound therapy. Open repair has the danger of scar dehiscence owing to poor skin conditions. In contrast, small invasive operations have the risk of sural nerve damage and a higher possibility of re-rupture. The gold standard method or approach is still under question. Plantar flexion in the ankle is primarily a function of the Achilles tendon; hence, post-operative plantar flexion is a significant determinant of the desired result. We present the case of a 57-year-old male farmer suffering from a left Achilles tendon rupture due to trivial trauma. This rupture consisted of a significant defect, present in the watershed area with signs of tendinosis at the insertion of the tendon. The patient was managed surgically by turn-o-plasty and the degenerated insertion site was augmented with the help of a suture disc. This case report focuses on surgical management by turn-o-plasty for significant defects in the Achilles tendon by using a suture disc to augment the defect.

## Introduction

Tendo Achilles is the body's strongest and largest tendon [1]. The musculotendinous junction of the gastrocnemius and soleus muscles ends 15 cm proximal to the insertion location on the posterior calcaneum, forming the Tendo Achilles or Achilles tendon. Unlike most tendons, the tendon of the Achilles is not covered by the synovial sheath in the lower extremity but by a structure known as the paratenon, which is acknowledged as the tendon's outermost layer. The paratenon resists the enormous tensile force imposed on the tendons, and primarily consists of a rich, collagenous, sparse extracellular matrix of cells and vessels (tenocytes). Tendon injuries heal slowly due to hypocellularity and poor vascularity. Although it has been stated that the paratenon is responsible for a large amount of the tendon's blood supply, the paratenon is connected to the avascular deep fascia and, therefore, this notion is questionable [2]. The paratenon, which surrounds the Achilles tendon, may protrude from the underlying tendon as a result of a haemorrhage. The watershed band is a portion of the tendon with an unstable blood supply [3]. This location can be found 2-6 cm from the tendon's insertion. About 75% of Achilles tendon ruptures occur in the watershed region, according to studies. Because the watershed region's blood supply is unstable, it is more likely to degenerate with age, resulting in more frequent ruptures. According to studies, 10-20% of cases occur at the insertion site and 5-15% at the musculotendinous junction [3]. The strongest tendon in the human body is also the most commonly torn tendon [4].

An Achilles tendon rupture is caused by a combination of predisposing factors and modes of damage. Oral or topical steroids, antibiotics (fluoroquinolones), exercise-induced hyperthermia, pathologically deteriorated tendons, senile decrease in the tendons, and mechanical abnormalities of the foot are all factors that have been studied [5-6]. Acute rupture is more likely in men between the ages of 40 and 50 as a result of sports accidents. There are several therapeutic options for Achilles tendon repair, including conservative care with cast immobilization or customized boots, as well as surgical repair using an open or percutaneous method [7].

Surgical management is superior to conservative therapy for significant and complete deformities. According to reports, 80-100% of patients who underwent surgical treatment were able to resume all of their previous activities. Immediate weight-bearing and immobilization closer to neutral plantarflexion are intended to minimize atrophy and stiffness after Achilles tendon replacement, but they may overstress the repair. The incidence of different consequences, most of which are due to operational treatment, such as deep vein thrombosis (DVT), wound infection, nerve injury, and skin-related disorders, is also an important factor to consider when making therapeutic decisions.

## Case presentation

A 57-year-old male, a farmer by profession, came to the outpatient department with a history of weakness in the left foot and difficulty in walking for 15 days after an episode of a fall at home. He gave a history of a "pop" sound during the fall. He gave no history of steroid or antibiotic intake. The patient was weight-bearing at the time of presentation.

On physical examination, the skin over the tendon was normal with no evidence of swelling, or engorged vessels. There was no tenderness but a defect was felt in the tendon substance on palpation. Plantar flexion of the left foot was weak. The Thompson test and Matles test were positive.

**Figure 1:**
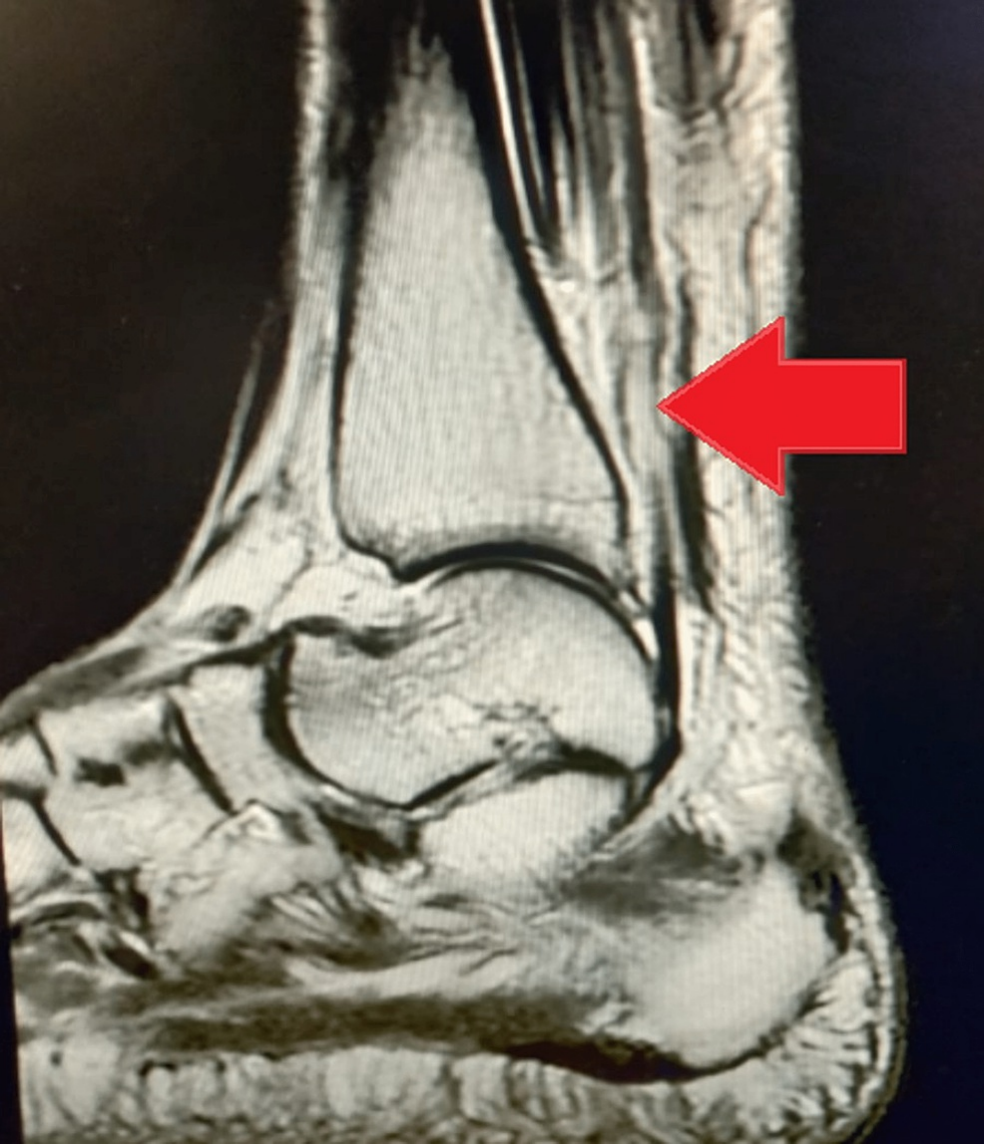
Pre-operative MRI showing large tendon defect (arrow)

Radiographic imaging showed no bony abnormality. Magnetic resonance imaging was performed and it was found that the tendo Achilles was ruptured with a defect of 4 cm and retraction of the torn end (Figure 1). Thus, the imaging findings correlated directly with the clinical examination. All the pre-operative assessments were done and the patient was planned for operation.

Surgical technique

**Figure 2:**
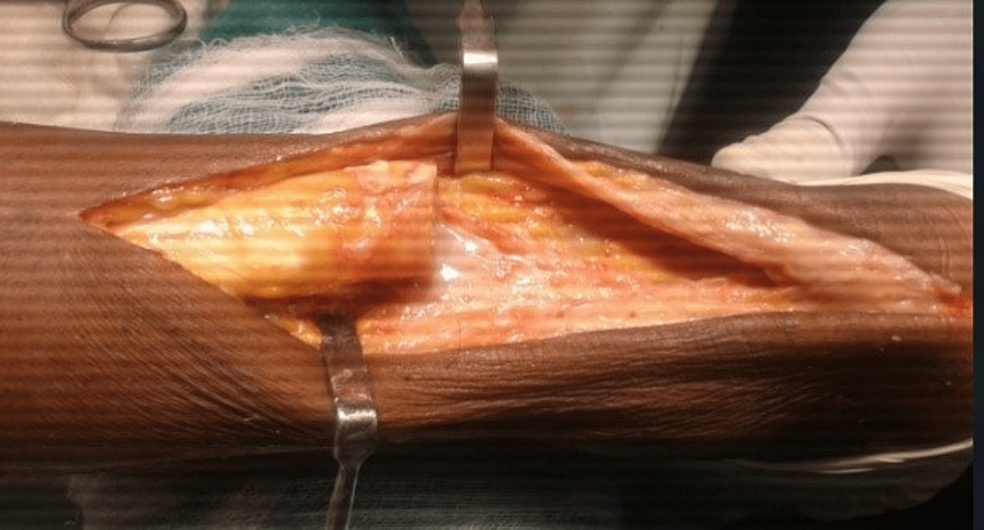
A defect of 7 cm seen in the substance of the Achilles tendon after freshening of the edges

The patient was taken to a prone position after giving spinal anesthesia, and painting and draping were done. A defect in tendon substance was identified by palpating and a 12 cm incision was made along the Achilles tendon on the posteromedial aspect of the leg. The short saphenous vein and sural nerve were meticulously identified and protected. On exposing the Achilles tendon, it was found that the distal part of the tendon at the insertion site has degenerated and calcification was present at the insertion site. The total defect calculated, after freshening the edges of the tendon, was 7 cm (Figure 2) and the distal part at the tendon insertion was not viable for end-to-end plasty of the tendon.

**Figure 3:**
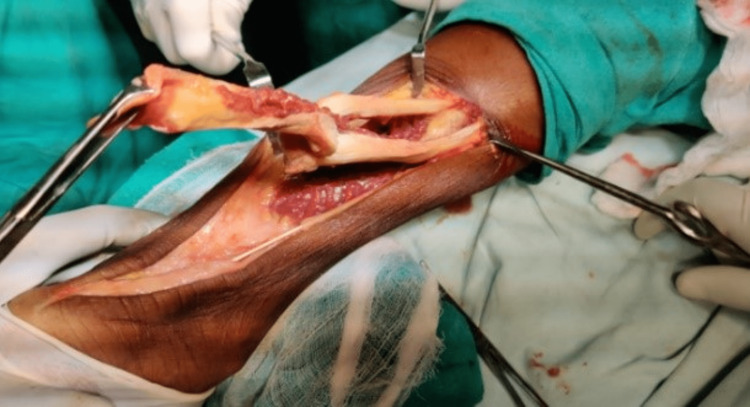
Turn-o-plasty in progress with the middle segment of the proximal tendon being reflected to complete the defect

**Figure 4:**
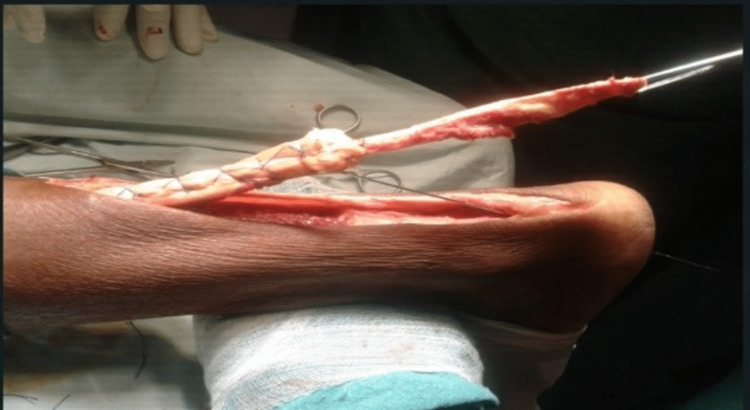
Augmentation of proximal remaining segment with Ethibond Ethibond^TM^ (Ethicon Inc, Raritan, New Jersey, United States)

**Figure 5:**
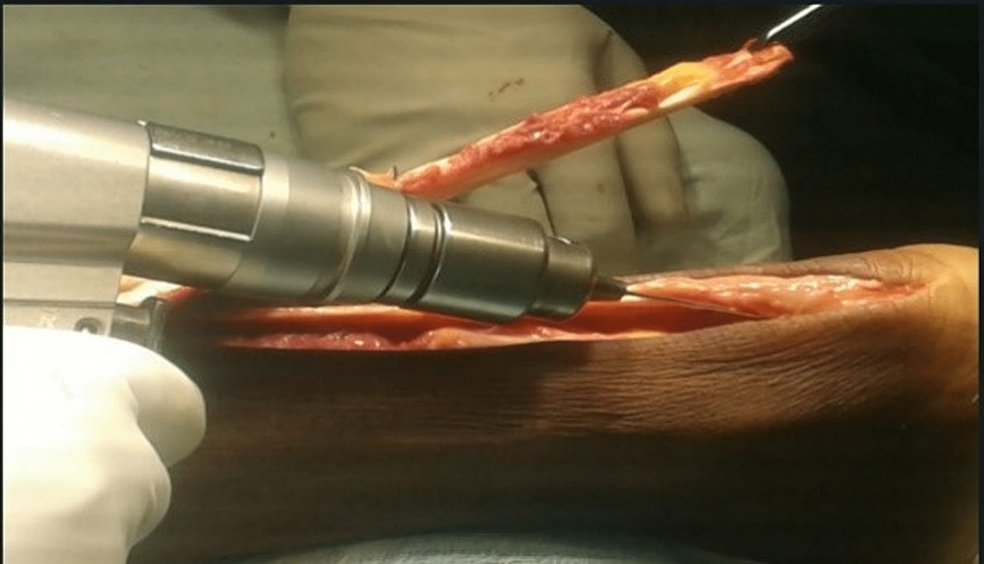
Showing a tunnel being created in the calcaneum by a K-wire. K-wire: Kirschner wire

The proximal part of the tendon was split in three and the middle portion was reflected (turn-o-plasty) and attached to the insertion site across the calcaneum after drilling through the calcaneum with a Kirschner wire (K-wire) and fixed between the lateral and medial process of the calcaneal tuberosity with the help of suture disc and Ethibond (Ethicon Inc, Raritan, New Jersey, United States) (Figure 3, Figure 4, and Figure 5).

Thompson's test and Matles test were found to be negative during on-table examination. Skin closure was done taking precautions not to induce tension over the skin. The dressing was done and a below-knee slab was applied and the patient was shifted to the post-operative recovery ward.

Post operation

**Figure 6:**
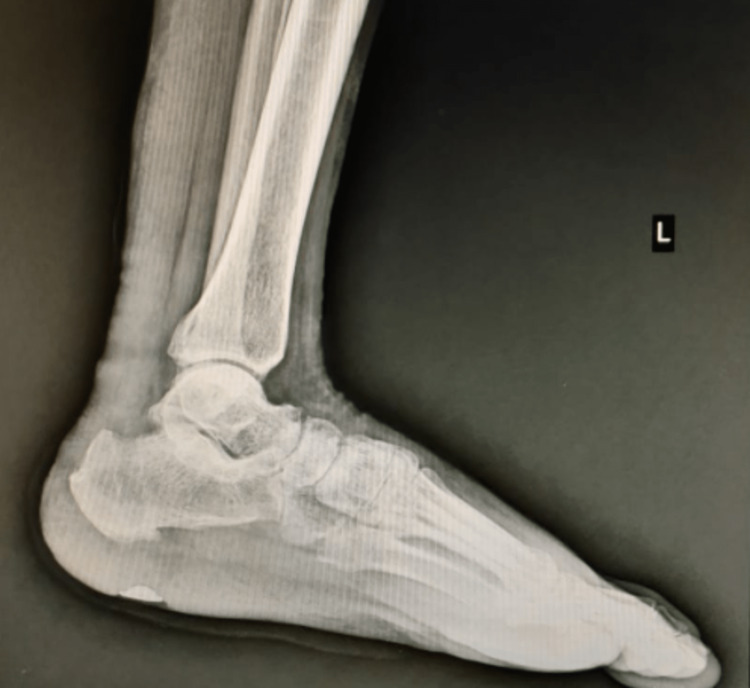
Post-operative x-ray showing suture disc

A post-operative x-ray was done and the suture disc was found to be in position (Figure 6).

**Figure 7:**
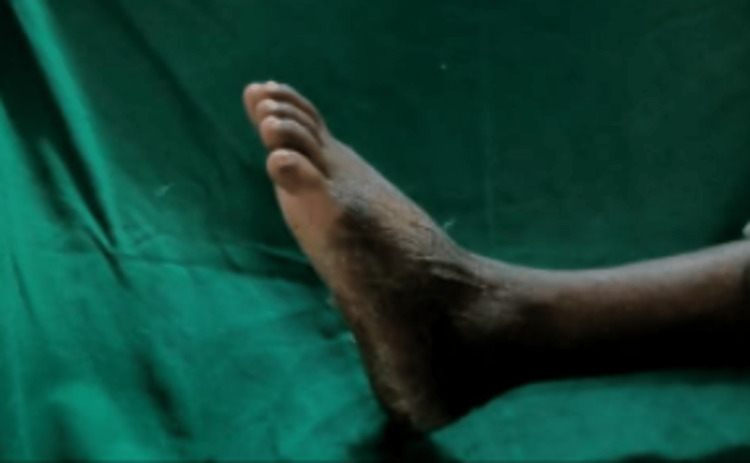
Post-operative plantar flexion

**Figure 8:**
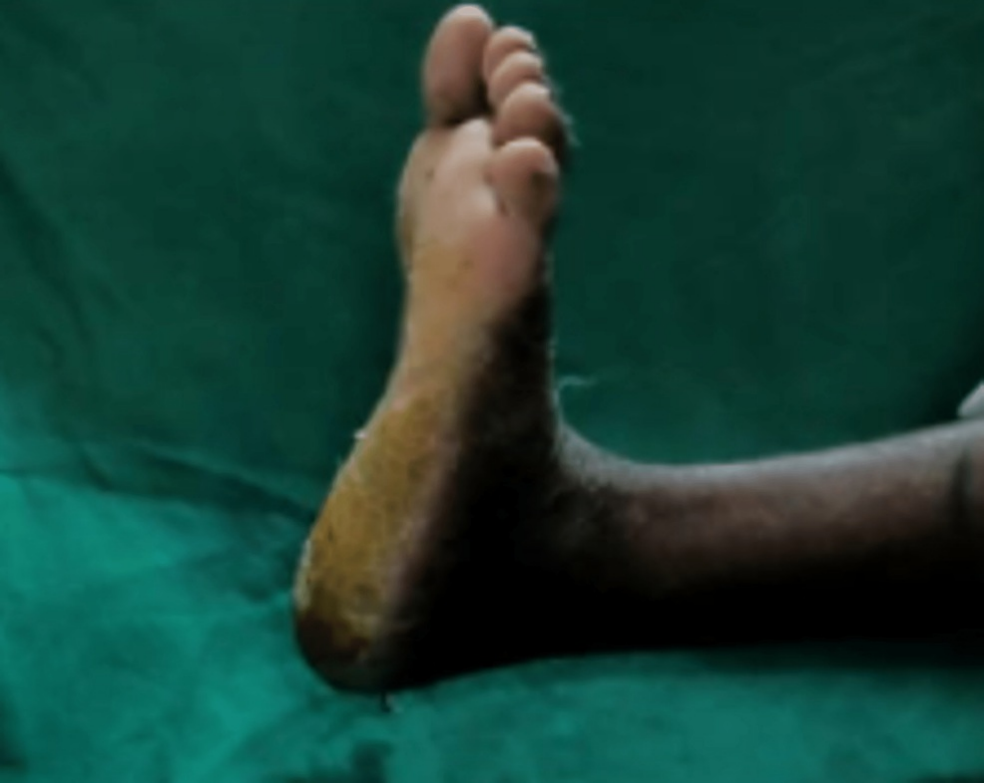
Post-operative dorsiflexion

The patient was given a below-knee slab in gravity equinus position and was converted to below-knee cast on the third day after check dressing and was kept non-weight bearing for four weeks. On the one-month follow-up, the below-knee cast and suture disc were removed. Thompson's text and Matles tests were negative and the ankle range of movement on the left side was 0-10 degree dorsiflexion and 0- 15 degree plantar flexion and same as the right side (Figure 7 and Figure 8).

The patient was started on gentle ankle range-of-movement exercises and he was given a heel raise of 6 cm on the left side and was advised to start progressive weight bearing and was advised not to squat. On the three-month follow-up, the patient regained his normal function and was able to squat without any difficulty. On the six-month follow-up, the patient was able to perform his daily activities without any difficulty and was able to do his work with full efficiency.

## Discussion

Much research on the various treatment techniques for Achilles tendon rupture has been published in recent decades. Nistor's biggest randomised prospective trial comprised 105 patients and found that 4% of surgically treated individuals had re-ruptures compared to 8% of conservatively managed patients [8]. Prior to surgery, Thompson's test and examination for palpable tendon and magnetic resonance imaging anomalies were used to confirm the diagnosis in all patients. Patients who were treated surgically showed earlier mobilization and a lower likelihood of reproduction than those who were treated conservatively. The exact cause of Achilles tendon rupture is yet unknown. The two most extensively contested ideas are chronic tendon degeneration and inhibitory system failure [9]. The first is supported by gross surgical and histological findings in the chronic degeneration tendon, which generally occur 2-6 cm above the os calcis. In their meta-analysis, Jiang et al. found that surgical therapy reduces the chance of re-rupture more effectively than conservative treatment, but it also raises the risk of complications. An increased risk of problems has been related to open repair surgery. However, current research does not give enough evidence to support the assumption that surgery may promote functional recovery [10]. In addition, Mullaney et al. found that following Achilles tendon replacement, there is disproportionate weakening in end-range plantar flexion and reduced passive stiffness in dorsiflexion. Anatomical lengthening, greater tendon compliance, and poor rehabilitation after Achilles tendon replacement are all possibilities [11]. Impairments will have an impact on activities (eg, descending stairs and landing from a jump). It's possible that end-range plantar flexion weakness after Achilles tendon surgery goes undiagnosed. Akizuki et al. demonstrated the usefulness of heel raise following Achilles tendon repair, claiming that a 1 inch heel raise was adequate to minimize plantar flexor activity during walking [12].

In their cohort, Westin and colleagues investigated the utility of ultrasound in determining the risk of re-rupture following Achilles tendon restoration and attempted to split patients into surgical and non-surgical treatment candidates based on pre-operative ultrasonography. If the diastasis between the tendon ends is more than 5 mm, surgical treatment was indicated to enhance clinical results [13]. Kotnis et al. have already shown this [14].

Young et al. performed a study to show that injecting organized collagen-containing mesenchymal stem cells into large tendon lesions may significantly improve the biomechanics, structure, and presumably function of the tendon after damage [15]. The use of platelet-rich plasma may also help in Achilles tendon repair.

## Conclusions

Turn-o-plasty is an effective approach for big tendon repair because it enables the surgeon to restore the tendon's original length without creating additional stress during the surgery. A suture disc is a successful implant utilized in the ligamentous repair of the knee. In this case report, we attempted to employ suture disc in the same way, with encouraging outcomes. The calcaneal fixation operation is technically hard but anatomically sound. The patient regained his full movements and was able to go back to his farming activities, reducing his financial constraints and allowing him to resume his life as before. Further research is needed to establish a suture disc as a feasible therapy option.
